# A5 USING AIR-LIQUID INTERFACE ORGANOID MONOLAYERS TO DEFINE MUCOSAL-MICROBE DYSFUNCTION IN PATIENTS WITH ULCERATIVE COLITIS

**DOI:** 10.1093/jcag/gwae059.005

**Published:** 2025-02-10

**Authors:** A Gilliland, Y Chen, I Ng, X Han, I Kalisky, H Sham, W Xiong, B Bressler, B vallance

**Affiliations:** The University of British Columbia, Vancouver, BC, Canada; The University of British Columbia, Vancouver, BC, Canada; The University of British Columbia, Vancouver, BC, Canada; The University of British Columbia, Vancouver, BC, Canada; The University of British Columbia, Vancouver, BC, Canada; The University of British Columbia, Vancouver, BC, Canada; The University of British Columbia, Vancouver, BC, Canada; The University of British Columbia, Vancouver, BC, Canada; The University of British Columbia, Vancouver, BC, Canada

## Abstract

NOT PUBLISHED AT AUTHOR’S REQUEST

Please acknowledge all funding agencies listed below

**Funding Agencies**: CCC, CIHRWeston Family Foundation

